# Ultra-high-resolution imaging of acute macular neuroretinopathy: a case report

**DOI:** 10.1016/j.ajoc.2026.102564

**Published:** 2026-03-18

**Authors:** Simon Magnin, Eva C. De Oliveira Figueiredo, Sandra Vermeirsch, Andrea Montesel, Chiara M. Eandi

**Affiliations:** a Department of Ophthalmology, University of Lausanne, Fondation Asile des Aveugles, Jules Gonin Eye Hospital, Lausanne, Switzerland; b Department of Surgical Sciences, University of Torino, Torino, Italy

**Keywords:** Adaptive optics, Imaging, Macula, Photoreceptor, Retina, Acute macular neuroretinopathy

## Abstract

We report the case of a 38-year-old woman presenting with acute bilateral paracentral scotomas in the context of a COVID-19 infection. Multimodal imaging confirmed the diagnosis of acute macular neuroretinopathy (AMN). Flood-illumination adaptive optics (FlAO) imaging was used to monitor photoreceptor structural anatomy over an 11-month period, providing high-resolution longitudinal data. FIAO analysis revealed a marked reduction in cone reflectivity in the affected areas at baseline, followed by partial restoration of the cone mosaic at follow-up, likely reflecting outer segment recovery.

To date, only few reports have described the use of adaptive optics in AMN with extended follow-up. This case highlights the utility of advanced imaging techniques in characterizing the evolution of pathologies such as AMN and monitoring their progression over time.

## Highlights

• FIAO imaging revealed reduced cone reflectivity with partial mosaic recovery at 11 months.
• FIAO findings spatially corresponded to EZ and ONL damage on SD-OCT.
• Cone density increased by 28%, consistent with outer segment recovery.
• FIAO enables in vivo photoreceptor-level monitoring beyond conventional imaging.

## 1 Introduction

Acute macular neuroretinopathy (AMN) is a rare retinal disorder first described by Bos and Deutman in 1975.^1^ AMN commonly affects young to middle-aged women and manifests as sudden-onset paracentral scotomas which may occur bilaterally.^2^ This condition is characterized by the presence of reddish-brown wedge-shaped parafoveal lesions oriented toward the fovea observed during fundus examination.^2^

While the exact pathophysiology remains uncertain, ischemic injury to the deep retinal capillary plexus and/or the choriocapillaris is considered the most plausible mechanism.^2^, ^3^, ^4^, ^5^ Several systemic and local factors have been associated with AMN, including viral infections, oral contraceptive use, and exposure to vasoconstrictive substances.^2^ Since the onset of the COVID-19 pandemic, reports of AMN have become more frequent, particularly in association with COVID-19 infection^6^^,^^7^ and vaccination.^8^^,^^9^

The diagnosis is based on clinical presentation and multimodal imaging. Near-infrared reflectance (NIR) imaging reveals sharply demarcated wedge-shaped hyporeflective lesions. Spectral-domain optical coherence tomography (SD-OCT) reveals corresponding hyperreflectivity or thinning of the outer nuclear layer (ONL) and disruption of the ellipsoid zone.^2^ Adaptive optics (AO) imaging allows direct in vivo visualization of the cone photoreceptor mosaic at a cellular level. In AMN, AO has been utilized to identify areas of photoreceptor loss or disorganization.^10^, ^11^, ^12^, ^13^, ^14^, ^15^, ^16^ While earlier studies have documented photoreceptor abnormalities in AMN using AO,^10^, ^11^, ^12^, ^13^, ^14^, ^15^, ^16^ there remains a lack of longitudinal imaging data in the existing literature.

## 2 Case presentation

**Fig. 1:**
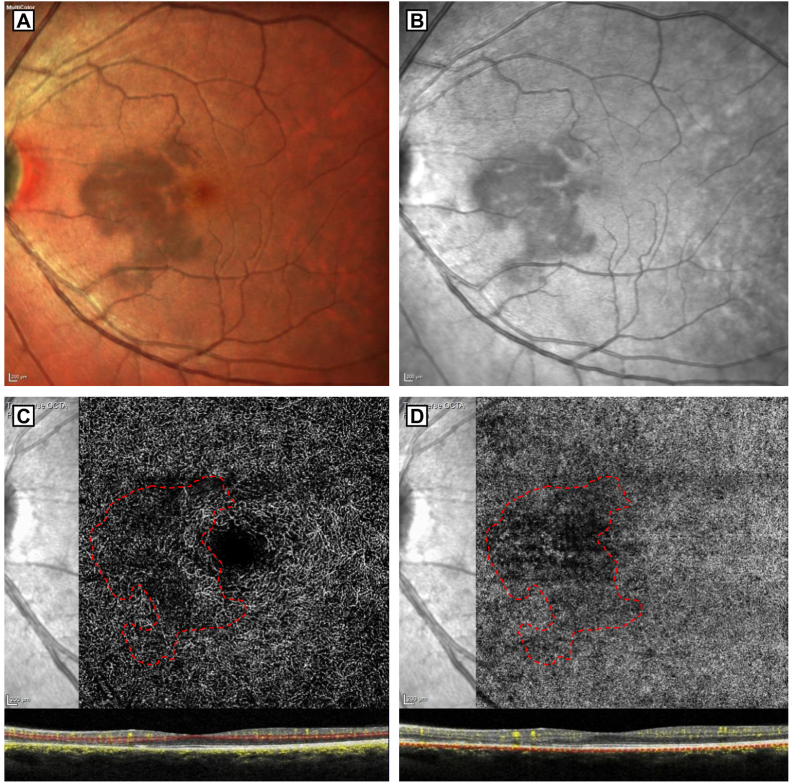
**Multimodal imaging of the left eye at initial presentation.** A) Multicolor Color fundus photograph showing reddish-brown, wedge-shaped parafoveal lesions. B) Infrared reflectance image revealing well-defined hyporeflective areas corresponding to the lesions.C-D) En face OCT-angiography of the deep capillary plexus (C) and choriocapillaris (D), showing localized flow attenuation corresponding to the area of ellipsoid zone disruption, which is outlined in red. (For interpretation of the references to color in this figure legend, the reader is referred to the Web version of this article.)

A 38-year-old Caucasian woman with no significant medical history presented with a two-week history of bilateral paracentral scotomas. Symptoms began concurrently with a mild COVID-19 infection, limited to fever without respiratory or systemic complications. She reported current use of oral contraceptives and had received COVID-19 vaccinations, the last dose ten months earlier. Best-corrected visual acuity was 20/20 in both eyes. Anterior segment examination was normal. Fundoscopy revealed reddish-brown, wedge-shaped parafoveal lesions in the nasal macula of both eyes (Fig. 1 A).

**Fig. 2:**
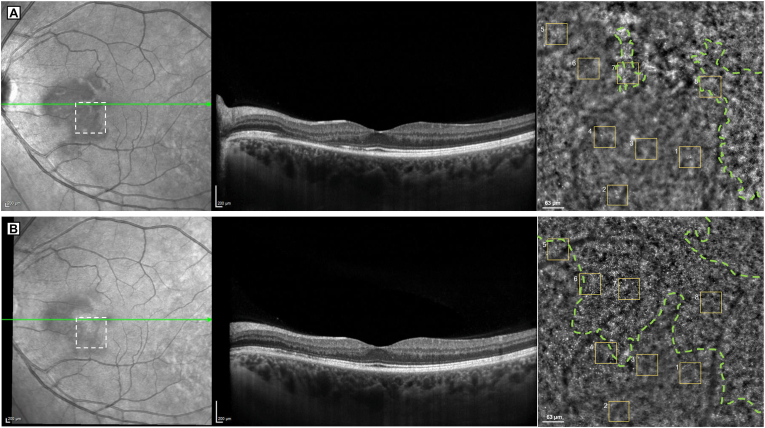
**Combined spectral-domain OCT (SD-OCT) and adaptive optics imaging of the left eye at baseline and 11-month follow-up.** A) At presentation, SD-OCT reveals disruption of the ellipsoid zone (EZ) with thinning of the overlying outer nuclear layer (ONL), consistent with acute macular neuroretinopathy (AMN). The white dashed square indicates the region shown at higher magnification in the adjacent adaptive optics (AO) image. AO reveals reduced cone density and marked disruption of the cone mosaic within the lesion, with the green dashed line outlining area where photoreceptor structure appears relatively preserved. Eight regions of interest (ROIs), each outlined in yellow (62 × 62 μm), were used for cone density analysis. B) At 11 months, SD-OCT demonstrates partial restoration of the EZ, although ONL thinning persists. AO image shows improved cone visibility across most ROIs, with an expansion enlargement of the area exhibiting preserved cone mosaic architecture (outlined in green), suggesting progressive structural recovery. (For interpretation of the references to color in this figure legend, the reader is referred to the Web version of this article.)

NIR imaging revealed well-demarcated symmetric bilateral hyporeflective paramacular lesions between the fovea and the optic disc (Fig. 1 B). Horizontal SD-OCT B-scans (Spectralis®, Heidelberg Engeneering, Heidelberg, Germany) showed thinning of the outer nuclear layer (ONL) associated with disruption of the ellipsoid zone (EZ) in both eyes, consistent with the diagnosis of AMN (Fig. 2 A). Short-wavelength autofluorescence revealed subtle hyperautofluorescence at the lesion site (not shown). Fluorescein and indocyanine green angiography were within normal limits. OCT-angiography (OCT-A) (Spectralis®) revealed localized attenuation of the deep capillary plexus (DCP) and choriocapillaris (Fig. 1C and D).

**Fig. 3:**
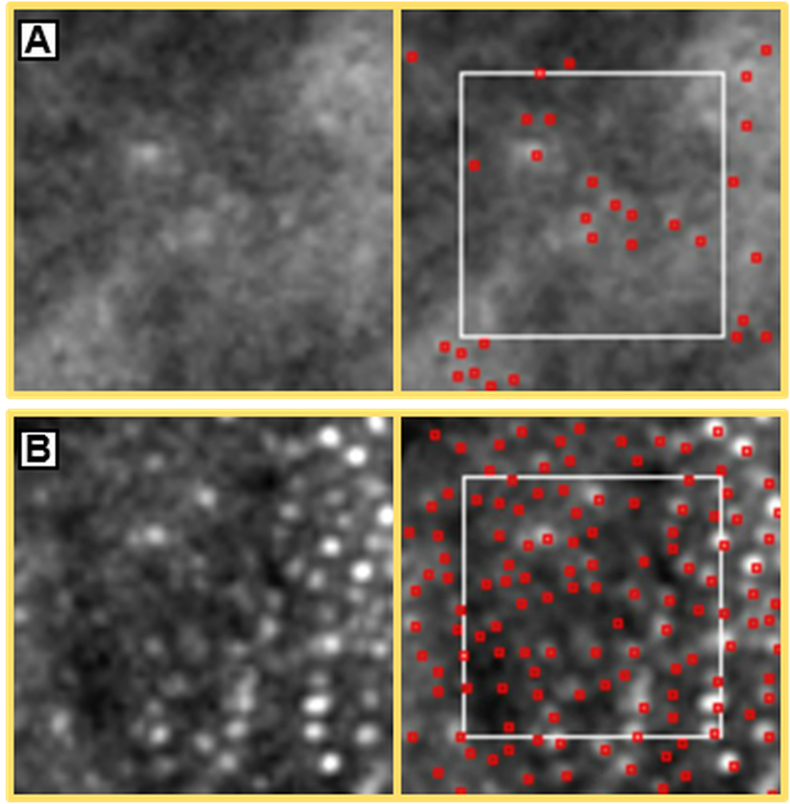
**Adaptive optics (AO) imaging of a selected region of interest (ROI) at baseline and 11-month follow-up.** Enlarged AO images of the ROI 6 (62 × 62 μm) at baseline (A) and 11 months (B). Each red dot corresponds to a cone identified by the automated segmentation algorithm (AODetect™). At baseline, photoreceptors are poorly distinguishable with disorganized and faint reflectivity. At 11 months, cone visibility and mosaic organization are notably improved. Cone density increased from 4710 cones/mm^2^ to 16,450 cones/mm^2^. (For interpretation of the references to color in this figure legend, the reader is referred to the Web version of this article.)

Flood-illumination AO (FlAO) imaging using the Rtx1 device (Rtx1, Imagine Eyes, France) was performed at 1 week and 11 months after presentation (Fig. 2, Fig. 3), with follow-up data available for the left eye only. At baseline, cone reflectivity was markedly reduced in the affected areas. Follow-up imaging at 11 months showed partial restoration of the cone mosaic, likely indicating recovery of the photoreceptor outer segment. This structural improvement corresponded with reduction in visual symptoms perceived by the patient and partial restoration of ellipsoid zone anatomy observed on SD-OCT.

Cone density was measured using AODetect™ automated segmentation tool in eight regions of interest (ROIs), each measuring 80 × 80 pixels (approximately 63 × 63 μm), within a 4° (1.2 × 1.2 mm) zone inside the lesion, including areas adjacent to the unaffected retina. The mean cone density increased from 11,670 cones/mm^2^ at baseline to 14,930 cones/mm^2^ at the 11-month follow-up. All cone density values for ROIs are summarized in Table 1.

**Table 1:** Cone density measurements in the eight regions of interest (ROIs) at baseline (1 week) and at 11-month follow-up. Each ROI corresponds to a defined area outlined in the adaptive optics (AO) images (Fig. 2). Cone densities varied across ROIs due to the inclusion of both affected and relatively preserved areas. For reference, reported values in healthy subjects at similar eccentricities (2–6°) are approximately 21,890 ± 1997 cones/mm^2^ nasally and 18,861 ± 1659 cones/mm^2^ inferiorly.^17^ All regions showed an increase in density over time, consistent with the structural improvements observed on AO imaging. Baseline measurement for region 3 could not be obtained due to insufficient cone visibility in the acute phase.

	Baseline (cone/mm^2^)	11 months (cone/mm^2^)	Difference (cone/mm^2^)
1	9810	11,470	+1660
2	10,200	10,430	+230
3	NA	11,050	NA
4	5000	13,850	+8850
5	13,630	14,910	+1280
6	4710	16,450	+11,740
7	15,420	17,730	+2310
8	22,890	23,580	+690

Mean	11,670	14,930	+3260

Values are expressed as cone density per mm^2^. Difference = 11 months – baseline. NA = not available.

## 3 Discussion

We described a case of AMN in a young woman in whom ultra-high-resolution imaging with FlAO revealed cone mosaic disruption in the acute phase and partial structural recovery after 11 months. This case underscores the unique ability of AO to asses in vivo photoreceptor-level changes that may not be detectable with conventional imaging, and might help furthering our understanding of the pathophysiology and recovery mechanisms in uncommon retinal disorders such as AMN.

The FlAO findings were consistent with other imaging modalities; areas of reduced photoreceptor reflectivity corresponded spatially to EZ disruption and ONL thinning on SD-OCT, as well as hyporeflective lesions on NIR imaging. These alterations reflect the typical layers affected in AMN and support the hypothesis of an ischemic injury at the interface between the retinal and choroidal circulations. The DCP, which supplies this watershed zone, is likely implicated in the pathogenesis. Ellipsoid zone involvement may result from reduced choriocapillaris perfusion or insufficient oxygen delivery from the DCP, which contributes approximately 10% of photoreceptor oxygen supply.^2^ In our case, OCT-A demonstrated localized perfusion loss in both the DCP and choriocapillaris in the area corresponding to the lesion. Previous studies have reported OCT-A abnormalities, involving the deep capillary plexus or the choriocapillaris 2, 3, 4, 5. Despite known artefacts on OCT-A, the spatial concordance across FlAO, OCT, and OCT-A findings supports a microvascular origin for outer retinal damage in AMN.

Quantitative analysis at baseline showed a mean cone density of 11,670 cones/mm^2^ across selected ROIs, markedly lower than reported values in healthy subjects: 21,890 ± 1997 cones/mm^2^ nasally and 18,861 ± 1659 cones/mm^2^ inferiorly at similar eccentricities (2–6°).^17^ The variability observed across ROIs likely reflects the fact that some of these were located at the lesion margins, where cone architecture appeared relatively preserved.

At 11 months, we observed a 28% increase in mean cone density (14,930 cones/mm^2^), consistent with previous AO studies reporting structural improvement over time.^11^, ^12^, ^13^, ^14^ However, Hansen et al.^10^ reported no changes at 6 months, suggesting that photoreceptor outer segment recovery or its detectability may be variable.

The observed increase in cone density in our case may reflect a combination of true outer segment recovery and improved cone reflectivity in regions that were only partially affected. In fact, cone visibility in AO imaging is influenced by photoreceptor orientation relative to the pupil, a phenomenon known as the Stiles-Crawford effect^18^^,^^19^; consequently, misaligned cones may appear hyporeflective or even undetectable. Bottin et al. further demonstrated that cone visibility in AMN can vary significantly depending on pupil entry angle, leading to substantial discrepancies in measured cone density.^15^ In addition, flood-illumination adaptive optics systems have limited ability to reliably resolve rod photoreceptors, which are smaller and exhibit lower reflectivity than cones.^20^ Therefore, although cones are the primary contributors to the imaged photoreceptor mosaic, we cannot fully exclude the possibility that some rods interspersed among cones—particularly in regions of structural disruption—may have contributed to density measurements. These limitations should be considered when interpreting quantitative photoreceptor metrics derived from FlAO imaging.

Finally, although visual recovery in AMN varies between patients, the relationship between structural restoration and functional improvement remains incompletely understood. In the present case, anatomical improvement observed on FlAO and SD-OCT was accompanied by partial subjective visual recovery; However, localized functional assessments such as microperimetry or multifocal electroretinogram were not available to allow precise structure-function correlation. Future studies combining adaptive optics imaging with spatially resolved functional testing, as previously reported by Audo et al.,^14^ may provide deeper insights into the functional significance of photoreceptor-level changes in AMN.

## 4 Conclusion

This case illustrates the clinical utility of AO imaging in diagnosing and monitoring AMN. Longitudinal imaging over 11 months revealed not only initial disruption of photoreceptor outer segments, but also signs of structural improvement, either due to outer segment recovery or improved photoreceptor orientation. These findings support the growing role of AO not only in the management of retinal disease, but also in advancing our understanding of its cellular-level pathophysiology.

## 5 Sex and gender bias reporting

In this case report, sex denotes biological attributes. Data on sex and gender were obtained as self-reported by the patient.

## CRediT authorship contribution statement

**Simon Magnin:** Writing – original draft, Visualization, Conceptualization. **Eva C. De Oliveira Figueiredo:** Writing – review & editing, Investigation. **Sandra Vermeirsch:** Writing – review & editing, Investigation. **Andrea Montesel:** Writing – review & editing. **Chiara M. Eandi:** Writing – review & editing, Supervision, Resources, Conceptualization.

## Patient consent

Written informed consent for publication was obtained. This report contains no personally identifiable information.

## Funding

This research did not receive any specific grant from funding agencies in the public, commercial, or not-for-profit sectors.

## Declaration of competing interest

The authors declare that they have no known competing financial interests or personal relationships that could have appeared to influence the work reported in this paper.
